# Advanced electrochemical biosensing of pathogens: Harnessing the antimicrobial properties of Ib-M peptides for highly sensitive bacterial detection

**DOI:** 10.1371/journal.pone.0337227

**Published:** 2025-11-21

**Authors:** J.L. Ropero-Vega, Y.J. Galvis-Curubo, J. M. Flórez-Castillo

**Affiliations:** 1 Facultad de Ciencias Exactas y Naturales, Universidad de Santander, Bucaramanga, Santander, Colombia; 2 Universidad del Magdalena, Santa Marta, Magdalena, Colombia; Islamic Azad University, IRAN, ISLAMIC REPUBLIC OF

## Abstract

This study describes the development of electrochemical biosensors with high sensitivity to detect pathogenic bacteria, including *Escherichia coli* O157:H7, *Pseudomonas aeruginosa*, and *Staphylococcus aureus*, in aqueous environments. The biosensors employ the antimicrobial peptides Ib-M1 and Ib-M6 as biorecognition elements, immobilized on gold nanoparticle-modified screen-printed electrodes via a self-assembled monolayer. Detection was achieved through electrochemical impedance spectroscopy, achieving remarkably low limits of detection of 1.4 CFU/mL for *E. coli* O157:H7 and *S. aureus*, and 0.8 CFU/mL for *P. aeruginosa*. The biosensors exhibited linear detection ranges of 0–100 CFU/mL for *E. coli* O157:H7 and *S. aureus*, and 0–75 CFU/mL for *P. aeruginosa*. Notably, the incorporation of carbon nanotubes significantly improved analytical sensitivity of the biosensors, particularly for *E. coli* O157:H7 and *S. aureus*. These results highlight the potential of the proposed biosensors for rapid, on-site monitoring of microbial contamination in drinking water, food processing environments, and clinical settings.

## Introduction

Waterborne diseases are a significant global concern, leading to severe health issues and, in some cases, fatal outcomes [1]. Pathogenic bacteria are among the primary agents responsible for these infections [2,3]. Timely detection of these microorganisms is crucial to ensure food safety and public health, as infections can be life-threatening without prompt treatment [4]. Among the most concerning pathogens are *Escherichia coli* O157:H7, which causes hemolytic uremic syndrome and hemorrhagic colitis, with even low concentrations potentially leading to kidney failure and death [5]. *Staphylococcus aureus* is another major pathogen, present in approximately 30% of the human population and associated with food poisoning through enterotoxin production [6,7]. In addition, *Pseudomonas aeruginosa* represents a critical threat as a multidrug-resistant Gram-negative bacterium capable of infecting multiple organs, especially in immunocompromised individuals [8,9].

Current detection methods such as culture techniques, polymerase chain reaction (PCR) and enzyme-linked immunosorbent assay (ELISA), are widely used due to their specificity and reliability [1,3,10]. However, during disease outbreaks, rapid and sensitive detection methods are essential. Electrochemical biosensors have emerged as a promising alternative for pathogen detection, offering rapid response times, high sensitivity, and the possibility of miniaturization and integration into portable platforms [11–13]. In addition, they are compatible with automation, require relatively simple instrumentation compared to conventional analytical methods, and can be adapted for on-site monitoring in complex environments. These devices integrate a biorecognition element, which provides specificity toward the target analyte, and a transducer, which converts the biochemical interaction into an electrical signal [14].

Electrochemical transducers are widely favored for their simplicity, cost-effectiveness, and accessibility [15]. Also, the incorporation of nanoparticles such as gold nanoparticles (AuNPs) enables the immobilization of biomolecules via thiol groups, enhancing sensor performance. On the other hand, carbon nanotubes (CNTs) are materials that possess exceptional electrical and mechanical properties, which facilitate rapid electron transfer and increase the sensitivity of the system. However, the transducer alone does not exhibit specificity, and it is the choice of bioreceptor that ensures the desired affinity of the assay [16].

Antimicrobial peptides (AMPs) have gained attention as bioreceptors due to their high affinity, stability, and cost-effectiveness [17–19]. These amphipathic peptides interact with bacterial membranes through electrostatic and hydrophobic interactions, making them ideal for bacterial detection. In 1997, Tailor *et al*. isolated four closely related antimicrobial peptides (Ib-AMPs) from *Impatiens balsamina* seeds [20]. Among these peptides, Ib-AMP4 has been extensively studied for its efficacy against various fungi, particularly filamentous fungi, and has demonstrated broad antibacterial activity against certain bacterial species. Subsequently, Flórez-Castillo *et al*. developed a series of peptides rationally derived from Ib-AMP4 by modifying the carboxyl terminal domain, replacing cysteine residues with arginine or tryptophan residues to alter their net charge and hydrophobicity [21]. These peptides, known as the Ib-M family, exhibit considerable improvement in the antimicrobial activity, structural stability, and ease of synthesis compared to their natural analogues. Initial findings suggest that the mechanism of action involve electrostatic and hydrophobic interactions between the peptides and bacterial membranes [22–27].

The Ib-M1 and Ib-M6 peptides of the Ib-M family are notable for their potent bactericidal action against *Escherichia coli*, including pathogenic strains such as O157:H7, with minimum inhibitory concentrations (MICs) ranging from 1.6 to 12.5 μM [24]. This efficacy is attributed to their ability to interact with the outer membrane of Gram-negative bacteria, a key feature for capturing biosensors based on biorecognition elements. Furthermore, both Ib-M1 and Ib-M6 adopt α-helical structures in hydrophobic environments, facilitating their orientation and stable anchoring on functionalized surfaces, such as those of modified electrodes [26]. This structural property improves immobilization efficiency and the exposure of key residues for interaction with the bacterial cell. Unlike many natural or synthetic antimicrobial peptides that require disulfide bonds, post-translational modifications, or long sequences, Ib-M peptides were designed without disulfide bonds and with short sequences (20 amino acids), which simplifies their chemical synthesis and increases their stability during the biosensor immobilization and storage process. Their rational design allows the incorporation of functional groups such as amino or thiol, to facilitate their covalent bonding to functionalized [23] or conductive surfaces, which is essential for achieving reproducible electrochemical sensors with good sensitivity.

Together, the properties of Ib‑M1 and Ib‑M6 peptides make them ideal candidates for use as biorecognition elements in the detection of pathogenic bacteria such as *E. coli*, *S. aureus* and *P. aeruginosa*, with potential applications in water quality monitoring or clinical diagnostics. The primary role of Ib-M peptides lies in the selective interaction with bacterial membranes through electrostatic and hydrophobic interactions, which initiates an impedimetric response. Therefore, the objective of this study was to develop and evaluate electrochemical biosensors based on Ib-M1 and Ib-M6 peptides for the sensitive detection of pathogenic bacteria, assessing their analytical performance under controlled laboratory conditions. These peptides were modified with cysteine at the amino terminus and immobilized on gold nanoparticle-modified screen-printed electrodes via Au-S bonds. Additionally, the incorporation of CNTs was evaluated to enhance the sensitivity of the biosensors for pathogenic bacteria detection.

## Materials and methods

### Reagents, Materials, and Instruments

All the chemicals were used as received without any further purification steps: potassium hexacyanoferrate (III) (K_3_[Fe(CN)_6_], purity above 99%, Merck), potassium hexacyanoferrate (II) (K_4_[Fe(CN)_6_]×3H_2_O, purity above 98%, Merck), potassium chloride (KCl purity above 99%, Sigma Aldrich, Burlington, MA, USA), sodium chloride (NaCl, PanReac), monopotassium phosphate, dipotassium phosphate (both procured from Sigma Aldrich), tetrachloroauric acid (HAuCl_4_·3H_2_O) (99.9%, Sigma Aldrich), sulfuric acid (98%, PanReac). The experiments were performed with Type–I water with a resistivity of 18.0 MΩ·cm^-1^. Multi-walled carbon nanotubes (CNT), functionalized with carboxylic acid and with a functionalization degree greater than 8%, were purchased from Sigma Aldrich.

Screen-printed electrodes (SPEs) were purchased from Italsens. These electrodes contain a carbon working electrode (WE) with a diameter of 3.0 mm, a carbon counter electrode (CE), and a silver/silver chloride (Ag/AgCl) pseudoreference electrode. In the present investigation, all the working potentials are reported with respect to the Ag/AgCl reference electrode. The electrochemical assays of the biosensors were performed at room temperature (25 °C) in a VersaSTAT3 (*Princeton Applied Research*) potentiostat/galvanostat/EIS analyzer connected to a computer and controlled by the VERSASTUDIO 2.60.6 software.

The antimicrobial peptides used were purchased commercially from Biomatik and were previously evaluated in antimicrobial activity tests against *E. coli* and some clinical isolates [21,26]. The sequences are presented below: peptide C-Ib-M1 sequence CEWGRRMMGRGPGRRMMRWWR-NH_2_, and peptide C-Ib-M6 sequence CEWGRRMMGWGRGRRMMRRWW-NH_2_. A cysteine was added to the N–terminal to induce the formation of stable S-Au bonds with the AuNPs on the surface of the biosensors through chemisorption [28].

The strains ATCC 43888 *Escherichia coli* O157:H7, ATCC 25923 *Staphylococcus aureus*, and ATCC 27853 *Pseudomonas aeruginosa* were stored at a cryogenic temperature of −80 °C in Luria Bertani (LB) broth supplied by Merck, with a 15% of glycerol content. To revive the bacteria, 50 µL of the frozen sample was introduced into 5 mL of LB broth and then incubated at 35 ± 2 °C for a period ranging from 18 to 24 hours prior to each test. The bacterial concentration was adjusted to 1 × 10^8^ CFU/mL using a saline solution with a concentration of 0.9%w/w [10,25,29]. The concentration of the bacteria to be evaluated in each test with the biosensors was established by means of serial dilutions of this solution and verified by means of the plate count technique. The bacterial count was determined starting from a concentration of 1 × 10⁸ CFU/mL, adjusted to a turbidity equivalent to 0.5 on the McFarland scale or an absorbance of 0.08 at a wavelength of 625 nm using spectrophotometric measurements. Subsequently, a 1:200 dilution was performed to achieve a concentration of 5 × 10⁵ CFU/mL, followed by a final dilution of 1:1000. Surface sowing was carried out from the final dilution. After 24 hours, the colony count was performed, which corresponded to an approximate value of 50 CFU [30].

### Preparation of electrochemical biosensors

Gold nanoparticles (AuNPs) were synthesized through electrochemical reduction in the following way: a 1.0 mM solution of tetrachloroauric acid (HAuCl_4_·3H_2_O) was prepared in 0.5 M sulfuric acid (H_2_SO_4_), serving as the supporting electrolyte. Then, 100 µL of this mixture was applied to the screen-printed electrodes (SPEs) and subjected to a potential of −0.05 V for 100 s. This specific potential was selected based on findings from our previous research [10,31]. The distribution of the synthesized AuNPs on the working electrode (WE) was examined using scanning electron microscopy. A Quanta Field Emission Gun microscope (Model 650) set to 15.0 kV was employed for this purpose, capturing images in secondary electron mode.

The peptides Ib–M1 and Ib–M6 were immobilized on the WE of the SPEs by forming an Au-S bond between the AuNPs and the N–terminal cysteine of the peptides. This was achieved by placing 10 µL of a peptide solution onto the WE and allowing it to sit for 16 h at an ambient temperature of about 25 °C. The peptide concentration was varied from 2.5 to 200 nM. Following chemisorption, the WE were washed with ultrapure water. The biosensors underwent electrochemical impedance spectroscopy (EIS) for characterization, both before and after each modification of the electrode, and they preserved their electrochemical performance for up to four weeks when stored dry at 4 °C. For the EIS measurements, 100 µL of a 5.0 mM hexacyanoferrate (II/III) solution in 0.1 M KCl was used as the redox probe on the surface of the biosensors. A frequency range of 50,000 to 0.5 Hz was scanned, applying 10 mV at open circuit potential (DC).

### Evaluation of the biosensors

The detection process consisted of placing the biosensors in a beaker containing 10 mL of a saline solution (NaCl 0.9% w/v, pH 7.2) with a predefined bacterial concentration ranging from 0 to 1000 CFU/mL [31]. The setup was then incubated at 25 °C for 20 min with continuous agitation at 150 rpm. Following incubation, the biosensors were carefully cleaned with ultrapure water before conducting electrochemical tests. Electrochemical impedance spectroscopy (EIS) measurements were carried out under identical conditions to those used during the initial characterization of the biosensors. Additionally, a SPE/AuNPs electrode without any peptide immobilization served as the control for the biosensors.

The interaction of bacteria with the surface of the working electrodes (WE) hinders electron charge transfer between the redox probe and the transducer. Consequently, the charge transfer resistance increases proportionally with bacterial concentration. The normalized resistance values (ΔR_Normalized_) were calculated from the EIS data using the following equation:


$${\Delta \text{R}}_{\text{Normalized}}=\frac{{\text{R}}_{\text{PEP}+\text{BACT}}-\;{\text{R}}_{\text{PEP}}\;}{{\text{R}}_{\text{PEP}}}$$ (1)

Here, R_PEP_ represents the charge transfer resistance of the electrode modified with peptides, and R_PEP+BACT_ denotes the charge transfer resistance of the biosensors when detecting bacteria. This approach allows to relate the charge transfer resistance of the biosensors to the bacterial concentration in the aqueous solution.

### Modification of biosensors with carbon nanotubes (CNTs)

Initially, CNTs were dispersed in a 1%w/v sodium dodecyl sulfate (SDS) solution, which served as a surfactant. The mixture, containing 1 mg of CNTs per mL, was subjected to ultrasonication in three 15-minute cycles, with 2 minutes of vortex mixing after each cycle. For deposition, 1.5 μL of the CNT suspension was drop-cast onto the working electrode, followed by drying at ambient temperature (25 °C) for 4 hours prior to the electrodeposition of gold nanoparticles (AuNPs). The preparation of the biosensors, including AuNPs deposition and peptide immobilization, was carried out as described previously.

## Results and discussion

### Preparation of the biosensors

**Fig 1:**
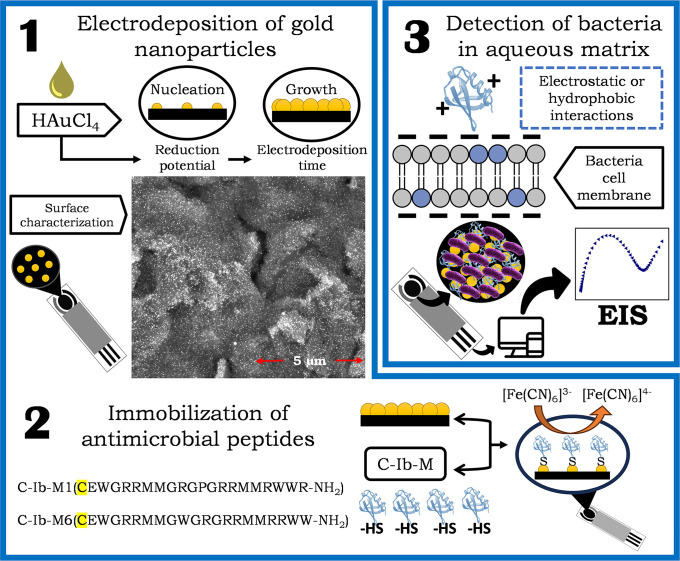
Schematic representation of preparation of biosensors. Electrodeposition of gold nanoparticles (1), immobilization of peptides (2), and mechanism for detecting microorganism (3).

The general outline of all steps involved in the preparation and evaluation of biosensors is illustrated in Fig 1.

In the first step, gold nanoparticles (AuNPs) were deposited on the electrode surface by electrochemical reduction of HAuCl_4_. This procedure resulted in homogeneous AuNPs coverage as shown in SEM micrography, improving both the conductivity of the system and the availability of active sites for subsequent functionalization. In the second step, synthetic peptides Ib-M1 and Ib-M6 were covalently immobilized on the AuNPs-modified surface. To this, both peptides were synthesized with a cysteine residue at the amino terminus, whose main function was to facilitate the formation of an Au-S bond between the thiol group (-SH) of the cysteine and the metallic surface of the AuNPs. This strategy ensures controlled orientation of the peptides within the transducer, which is essential to preserve their biological recognition capability and maximize the sensitivity of the system.

Finally, the detection principle is based on the hypothesis that peptides interact with target microorganisms through noncovalent forces, primarily hydrophobic and electrostatic interactions. These interactions induce changes in the electronic transport properties of the system, resulting in measurable variations in electrochemical signals such as the system impedance. These changes indicate the presence and, potentially, the concentration of the target microorganisms.

The C-Ib-M6 peptide was selected as an experimental model for the optimization phase of the preparation conditions of the electrochemical biosensors. This facilitates the clear identification of the individual effect of peptide concentration, ensuring greater experimental control and avoiding the complexity associated with the simultaneous evaluation of both peptides. Furthermore, previous studies have shown that C-Ib-M6 possesses a stable α-helical structure, good antimicrobial activity against *Escherichia coli*, and low cytotoxicity in eukaryotic cells, properties comparable to those of the C-Ib-M1 peptide [24,26]. These characteristics make C-Ib-M6 a representative candidate for the standardization of system conditions. Finally, this choice allowed for efficient optimization of peptide concentration and subsequently applying the standardized conditions to C-Ib-M1, with minimal adjustments.

**Fig 2:**
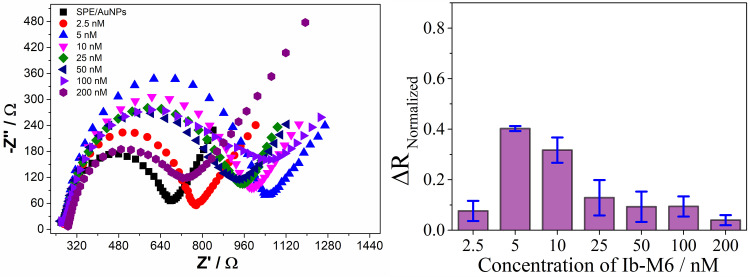
Nyquist diagram plots (left) and normalized resistance (ΔR_Normalized_, right) of the immobilization of C-Ib-M6 peptide at different concentrations on AuNPs-modified screen-printed electrodes.

The Nyquist diagrams shown in Fig 2 (left) reveal that the electrode modified with gold nanoparticles (SPE/AuNPs (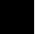)) exhibits a semicircular configuration. This indicates that the electrochemical activity is predominantly controlled by the electron transfer process, coupled with the inherent resistance of the surface of the electrode [10].

After modifying the electrodes with AuNPs, they were exposed to solutions of C-Ib-M6 (2.5, 5, 10, 25, 50, 100, 200 nM) to determine the optimal concentration for immobilizing the biological recognition element. The presence of the recognition element altered the surface of the working electrode. Analysis of the impedance spectra displayed in the Nyquist diagram in Fig 2 (left) reveals that the spontaneous bond formation (Au-thiol) through direct cysteine adsorption as the primary functionalization interface on the AuNPs disrupted the double charge layer at the electrode/electrolyte interface. This disturbance led to changes in capacitance and increased resistance to electron transfer [31,32]. These alterations in signals suggest that the peptide induced modifications in the ion diffusion phenomena between the electrolyte solution and the electrode, indicating surface changes [33].

Normalized charge transfer resistance values (ΔR_Normalized_, eq. 1) correlated with peptide concentration were calculated to determine the appropriate immobilization concentration. As can be seen in Fig 2 (right), an increase in peptide concentration from 2.5 to 5 nM considerably increases the ΔR_Normalized_ values, but the latter has no significant differences when the peptide concentration increases to 10 nM. A subsequent increase in peptide concentrations from 25 to 200 nM decreases the ΔR_Normalized_ values, probably due to electrostatic interactions between the peptide and the hexacyanoferrate redox probe, or to the formation of a saturated peptide layer that favors charge transfer in the solution. However, this decrease in resistance is unlikely to correlate with an expected increase in charge transfer resistance when the biosensors interact with bacteria.

**Fig 3:**
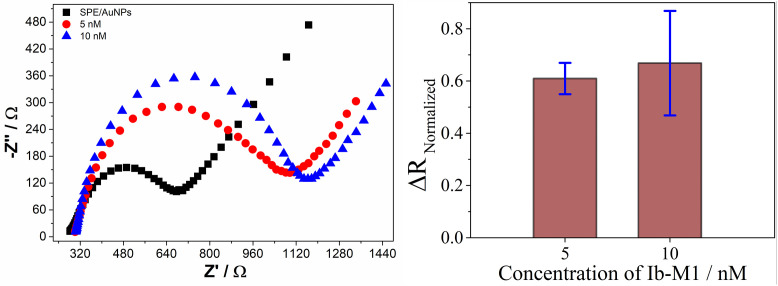
Nyquist diagram (left) and ΔR_Normalized_ values (right) in the evaluation of concentrations for the immobilization of C-Ib-M1 peptide.

Based on the results obtained for the C-Ib-M6 peptide, concentrations of 5 and 10 nM were selected to evaluate the behavior of the biosensors in the immobilization of the C-Ib-M1 peptide and the results are shown in Fig 3.

As expected, there is no significant difference in varying the peptide concentration between 5 and 10 nM for C-Ib-M1. Additionally, it is noteworthy that the ΔR_Normalized_ values at 5 nM is more stable across multiple measurement repetitions for both peptides. For this reason, the concentration of 5 nM was selected as the most appropriate for the microorganism detection tests.

### Electrochemical detection

**Fig 4:**
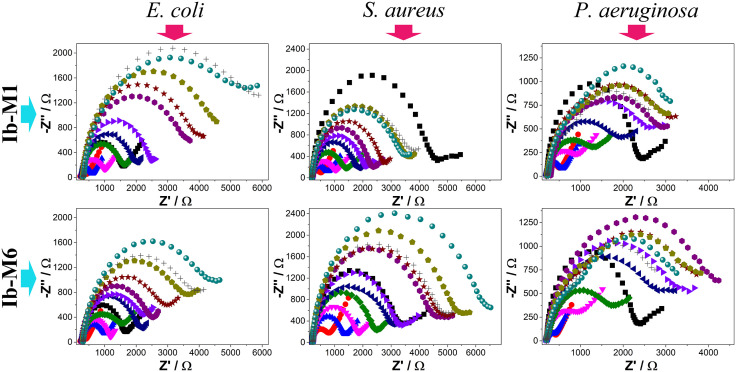
Nyquist diagrams of the detection of *Escherichia coli* O157:H7, *Staphylococcus aureus*, and *Pseudomonas aeruginosa* by using C-Ib-M1 and C-Ib-M6 peptides as recognition elements. SPE (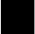), SPE/Au (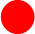), SPE/Au/Ib-M (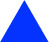), 0 (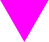), 10 (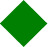), 30 (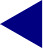), 50 (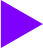), 75 (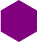), 100 (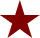), 200 (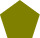), 500 (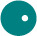), and 1000 (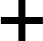) CFU/mL.

The biosensors were tested with three reference bacteria (*E. coli* O157:H7, *S. aureus*, and *P. aeruginosa*) and the C-Ib-M1 and C-Ib-M6 antimicrobial peptides as recognition elements, using an immobilization concentration of 5 nM. The biosensors developed were evaluated for detecting various concentrations (0, 10, 30, 50, 75, 100, 200, 500, and 1000 CFU/mL), showing a change in resistance proportional to charge transfer in the Nyquist diagrams as the bacterial concentration increased (Fig 4).

Based on the provided sources, the baseline signal at 0 CFU/mL depicted in Fig 4 slightly modifies the response of the biosensors (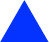), presumably because of interactions between ions and the surface, though the alteration is slight. Conversely, even at the minimal concentration evaluated (10 CFU/mL), the presence of bacteria markedly influences the electrical transport characteristics of the biosensors in comparison to the baseline. This phenomenon is attributed to the creation of a complex involving C–Ib–M peptides and bacteria, facilitated by electrostatic and hydrophobic forces, which leads to changes in the dielectric or conductivity attributes and consequently affects the rate of charge transfer [34,35]. The presence of a semicircle in the impedance spectra suggests a uniform distribution of time constants within the frequency range studied [36]. However, the presence of a large semicircle without a straight line indicates a slow electron transfer step [37]. The diameter of the semicircle corresponds to the charge transfer resistance (R_ct_) of the redox probe, which increases as more bacteria approach the surface [38,39].

The biosensors using the C-Ib-M1 peptide displayed a semicircle with an increasing diameter observed up to the highest concentration (1000 CFU/mL) for *E. coli* (Fig 4, top left). For *S. aureus*, no significant differences were noted at the highest concentrations of 200, 500, and 1000 CFU/mL (Fig 4, top center). Finally, the detection of *P. aeruginosa* showed the formation of two semicircles starting from a concentration of 50 CFU/mL at high frequencies, with an increase in semicircle diameter observed up to a concentration of 500 CFU/mL (Fig 4, top right). The shape of a Nyquist plot is influenced by the electrode matrix and the electrochemical responses occurring on the working electrode surface or in the redox probe [40]. Therefore, the above indicates that *P. aeruginosa* behaves differently compared to the other two reference bacteria.

The biosensors with peptide C-Ib-M6 showed an increase in the semicircle up to a concentration of 500 CFU/mL for *E. coli* and *S. aureus* (Fig 4, bottom left and middle, respectively). However, the detection of *P. aeruginosa* showed the formation of two semicircles from the concentration of 75 CFU/mL as it was observed with the biosensors with C-Ib-M1 (Fig 4, bottom right).

**Fig 5:**
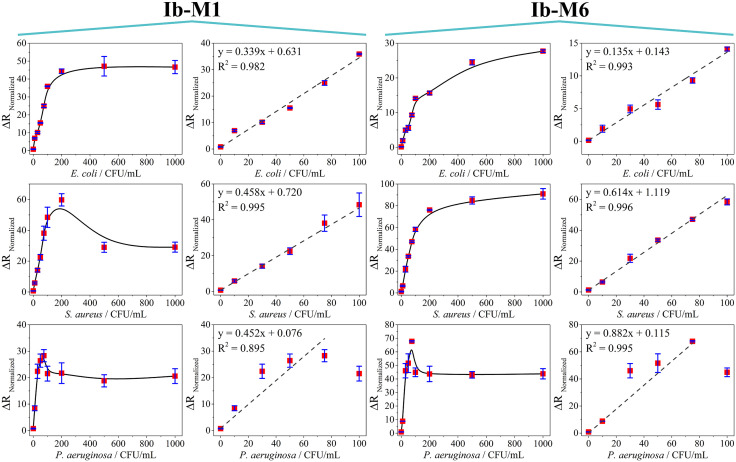
Calibration curves of the biosensors in the detection of *E. coli* (top), *S. aureus* (middle) and *P. aeruginosa* (bottom).

The Nyquist diagrams were adjusted based on the typical Randles circuit to determine the charge transfer resistance (R_ct_) and capacitance values in each measurement [41,42]. The Randles equivalent circuit comprises a solution resistance (R_sln_), constant phase element (CPE), charge transfer resistance (R_ct_), and Warburg impedance (W). R_sln_ and W reflect the fundamental properties of electrolyte and redox probe diffusion, while CPE and R_ct_ are influenced by dielectric and insulating characteristics at the electrode/electrolyte interface [43]. This analysis enabled the calculation of normalized charge transfer resistance values (ΔR_Normalized_, eq. 1) that were correlated with bacterial concentration in calibration curves as shown Fig 5.

The normalized charge transfer resistance values derived from the equivalent circuit modeling revealed a linear relationship with bacterial concentrations. In the electrochemical context, the essence lies in the hindrance of charge transfer of the redox couple from the solution to the electrode surface due to the interaction between the target bacteria and peptides. Nonetheless, the linear behavior varies depending on the specific bacterium and peptide used. Loss of linearity in the biosensors indicates saturation with bacteria under such circumstances.

The biosensors with C-Ib-M1 peptide exhibit linearity in the detection of *E. coli* and *S. aureus* from 0 to 100 CFU/mL, with R^2^ values of 0.982 and 0.995, respectively. In the detection of *P. aeruginosa*, linearity was adjusted from 0 to 100 CFU/mL, however, most of the points studied did not fit the model, resulting in a R^2^ value of 0.895 (Fig 5, bottom left). On the other hand, the biosensors incorporating the C-Ib-M6 peptide exhibited linear behavior for concentrations ranging from 0 to 100 CFU/mL in detecting *E. coli* and *S. aureus*, with R^2^ values of 0.993 and 0.996, respectively. The R^2^ values increased for both bacteria compared to the C-Ib-M1 biosensors, indicating better adjustment to linearity. While the detection of *P. aeruginosa* showed an improved R^2^, there were still points deviating from the linear model (Fig 5, bottom right).

The calculation of the limits of detection and quantification (LOD and LOQ) were performed using the following equation:


$$\text{LOD or LOQ}=k\cdot \frac{{\text{S}}_{\text{bl}}}{m}$$ (2)

Where *k* equals 3 for LOD, reflecting a 98.3% confidence interval, and 10 for LOQ. The term S_bl_ is the standard deviation of the blank sample (0 CFU/mL), while *m* is the slope of the calibration curve. Also, the analytical sensitivity, which represents the ability of the biosensors to distinguish small concentration differences considering noise, was calculated with the following equation:


$$\text{Analytical sensitivity}=\;\frac{m}{{\text{S}}_{\text{bl}}}$$ (3)

The parameters obtained from each constructed biosensor are listed in Table 1.

**Table 1:** Analytical parameters of the biosensors.

Analytical parameter	*E. coli*		*S. aureus*		*P. aeruginosa*	
	Ib-M1	Ib-M6	Ib-M1	Ib-M6	Ib-M1	Ib-M6
Linear Detection Range (CFU/mL)	0 - 100	0 - 100	0 - 100	0 - 100	0 - 50	0 - 75
Limit of Detection (CFU/mL)	1.4	1.5	4.3	1.3	0.8	1.9
Limit of Quantification (CFU/mL)	4.7	4.9	14.5	4.4	2.7	6.4
Analytical sensitivity (1/CFU·mL^-1^)	2.142	2.036	0.692	2.266	3.663	1.557

The detection of *E. coli* with both biosensors did not present major differences in LOD and LOQ values. However, a slightly higher value of the analytical sensitivity of the biosensors with C-Ib-M1 indicates that it could be better. On the other hand, the biosensors exhibited differences towards the detection of *S. aureus*. Although the linear detection range was the same, the biosensors with C-Ib-M6 exhibited low detection and quantification limits, as well as much higher analytical sensitivity compared to C-Ib-M1. Finally, low limits of detection were found for both biosensors in the detection of *P. aeruginosa*, but with smaller linear detection ranges compared to the other two microorganisms. The above indicates that the biosensors prepared with these peptides could be used in the detection of these pathogenic microorganisms in an aqueous matrix.

**Fig 6:**
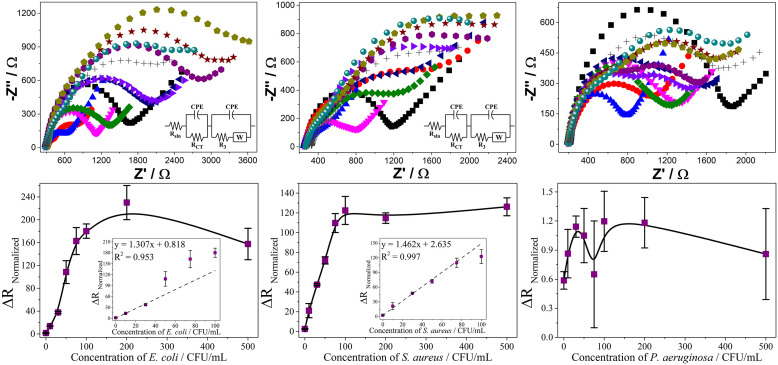
Electrochemical response of the CNTs-modified biosensors in the detection of *E. coli* (left), *S. aureus* (middle) and *P. aeruginosa* (right). Detection was studied by Nyquist diagrams (top) in a concentration range of 0 to 500 CFU/mL. SPE (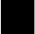), SPE/CNTs (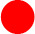), SPE/CNTs/Au (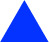), SPE/Au/Ib-M (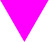), 0 (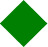), 10 (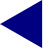), 30 (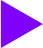), 50 (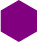), 75 (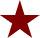), 100 (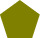), 200 (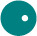), and 500 (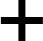) CFU/mL. Calibration curves (bottom) obtained for each biosensor.

Considering the satisfactory results obtained using C-Ib-M peptides as recognition elements, the biosensors were modified with carbon nanotubes (CNTs) to evaluate the possible improvement in the analytical characteristics of the devices. The purpose of CNTs deposition is to provide a higher surface area and electrical transport properties of the electrode, thereby improving the electrochemical response of the biosensors. This also contributes to more stable receptor immobilization and bioaffinity, ultimately increasing the efficiency of analyte detection. The dispersion conditions of the CNTs allowed the deposition on the screen-printed electrodes, each maintaining their electrochemical properties observed in the Nyquist diagrams (Fig 6). C-Ib-M1 peptide was used for the CNTs-modified biosensors in the detection of *E. coli* and *P. aeruginosa* while C-Ib-M6 was used for *S. aureus*. They were selected considering the best results obtained in analytical sensitivity (Table 1). For the calibration curves, an equivalent circuit model was used with two resistors and two constant phase elements (inserts in Fig 6).

All CNTs-modified biosensors were evaluated in a microorganism concentration range of 0–500 CFU/mL. For *E. coli* and *S. aureus*, a linear trend is observed up to 100 CFU/mL. Above this bacterial concentration, the biosensors do not respond to changes in concentration, suggesting system saturation. In the case of *P. aeruginosa*, biosensor responses were not observed towards the detection of the microorganism in the entire range of concentrations evaluated. This may be because this microorganism has been shown to be prone to aggregate into multicellular groups in the presence of low concentrations of CNTs, which enhances its biofilm formation capacity [44]. The formation of biofilms on the electrode surface is an undesirable process since it limits or eliminates the electrical and mass transport processes between the redox probe and the biosensor, making its application impossible.

The CNTs-modified biosensors exhibited satisfactory behavior at low concentrations with LOD and LOQ of 1.4 and 4.6 for *E. coli* and 0.7 and 2.3 for *S. aureus*. It is noteworthy that while the analytical sensitivity was 2.178 for *E. coli*, for *S. aureus* it reached 4.430, almost double that obtained without the use of CNTs. Likewise, the LOD and LOQ of *S. aureus* were reduced by half. Therefore, the integration of carbon nanotubes significantly improves both the interactions with the receptor and the transduction of electronic signals, providing a larger active surface area on the electrode which gives rise to improved electrochemical responses.

Conventional methods such as culture, PCR, and ELISA are well-established and widely accepted for bacterial detection, but they present drawbacks in terms of analysis time, cost, and instrumentation requirements [45]. In contrast, the proposed peptide-based electrochemical biosensors provide results in approximately one hour with detection limits comparable or even superior to those conventional methods, while using low-cost and portable equipment. This contrasts with other detection strategies that, although capable of achieving low detection limits, rely on conventional three-electrode electrochemical cells, making them more complex and less practical to implement [46]. These features make our approach attractive for decentralized monitoring of water quality, particularly in low-resource settings where rapid and affordable detection is crucial. A comparative summary is presented in Table 2, where the main analytical parameters of different methods are contrasted reported in the literature. The analysis shows that peptide-based electrochemical biosensors, particularly CNT-modified systems, combine competitive sensitivity and detection limits while retaining the aforementioned advantages.

**Table 2:** Comparison of electrochemical biosensors and conventional methods for bacterial detection.

Recognition element/ Method	Target microorganism	Detection principle	Typical LOD (CFU/mL)	Response time (h)	Cost	Reference
Culture (plate count)	Broad spectrum	Growth on selective media	~10	24-72	Low	[47]
PCR	Specific genes of *E. coli*, *S. aureus*, *P. aeruginosa*, etc.	Amplification of DNA	1-10	4-8	High	[45]
ELISA	Species-specific antigens	Antigen–antibody binding	10-100	4-6	Medium-high	[45]
Antibody- MWCNTs	*E. coli*	Voltammetry	26	1	Low	[48]
Antibody-SWCNT	*E. coli*	Current	100	5 min	Low	[49]
LecB protein	*P aeruginosa*	Electrochemical impedance	10	1	Low	[50]
Hybrid nanocomposites	*E. coli* O157:H7 *Salmonella typhimurium*	Voltammetry	25	< 1	Low	[51]
Polyclonal antibody/BSA protein	*E. coli*	Voltammetry	3.02	1	Low	[46]
C-Ib-M1 + CNTs	*E. coli*	Electrochemical impedance	~1	1	Low	This work
C-Ib-M6 + CNTs	*S. aureus*	Electrochemical impedance	~1	1	Low	This work
C-Ib-M1	*P aeruginosa*	Electrochemical impedance	~1	1	Low	This work

It is worth noting that the present results were obtained under controlled laboratory conditions, and the specificity of the system against non-target microorganisms and its robustness in complex matrices remain to be addressed. Antimicrobial peptides may interact with bacterial membranes in a broader manner, which could result in cross-reactivity, false positives in the presence of dead cells or cell debris, or false negatives under competitive binding in complex microbial backgrounds. Therefore, future work will focus on evaluating all these phenomena, as well as validating the biosensor in real environmental water samples. These efforts will be critical to advancing from the current proof-of-concept and validation (Technological Readiness Level, TRL 4) to higher readiness levels, ultimately enabling the translation of this technology to practical applications in water quality monitoring and clinical diagnostics.

## Conclusions

This research demonstrates the effective preparation and evaluation of electrochemical biosensors by using gold nanoparticles (AuNPs) and antimicrobial peptides (C-Ib-M1 and C-Ib-M6) for the detection of pathogenic microorganisms. The electrochemical detection mechanism relied on the interaction between the peptides and target microorganisms, leading to measurable changes in electronic transport properties, specifically charge transfer resistance.

The optimization of peptide concentration revealed that 5 nM was optimal for the performance of the biosensors, which was confirmed through impedance spectroscopy analyses. Furthermore, the biosensors exhibited linear detection ranges of 0–100 CFU/mL for *E. coli* and *S. aureus*, and 0–75 CFU/mL for *P. aeruginosa*, highlighting their potential applicability in pathogen detection within aqueous matrices. The incorporation of carbon nanotubes further enhanced the analytical sensitivity of the biosensors and reduced detection limits, specifically for *E. coli* and *S. aureus*, demonstrating the benefits of nanomaterial integration in biosensor technology. Overall, the findings underscore the promise of these biosensors as reliable tools for the rapid detection of harmful microorganisms, with implications for public health and safety.
